# Developing a Web-Based Asynchronous Case Discussion Format on Social Media to Teach Clinical Reasoning: Mixed Methods Study

**DOI:** 10.2196/45277

**Published:** 2023-08-09

**Authors:** Casey N McQuade, Michael G Simonson, Kristen A Ehrenberger, Amar Kohli

**Affiliations:** 1 Division of General Internal Medicine Department of Medicine University of Pittsburgh School of Medicine Pittsburgh, PA United States; 2 Department of Pediatrics University of Pittsburgh School of Medicine Pittsburgh, PA United States

**Keywords:** case discussion, case report, clinical reasoning, clinical vignette, junior doctor, junior physician, medical education, medical student, morning report, report style, resident, social media, trainee, Twitter

## Abstract

**Background:**

Case-based learning conferences are valuable to trainees, but growing clinical demands hinder consistent attendance. Social media increasingly acts as a venue for trainees to supplement their education asynchronously. We designed and implemented a web-based asynchronous clinical case discussion series on the Twitter social media platform to fill this educational gap.

**Objective:**

The aim of this mixed methods study is to examine the nature of interactions among web-based case discussion participants and assess local attitudes regarding the educational intervention.

**Methods:**

Starting in February 2018, we posted clinical vignettes to a dedicated Twitter account with the prompt “What else do you want to know?” to stimulate discussion. The authors replied in real time when case discussion participants requested additional details. Additional data about the case were posted at regular intervals to the discussion thread to advance the overall case discussion. Participants were asked to explain their reasoning and support their conclusions when appropriate. Web-based engagement was assessed using Twitter Analytics. Participants’ posts were qualitatively analyzed for themes, with special attention to examples of using clinical reasoning skills. A codebook of types of participant posts and interactions was refined iteratively. Local engagement and attitudes at our institution were assessed by surveying internal medicine trainees (n=182) and faculty (n=165) after 6 months.

**Results:**

Over a 6-month period, 11 live case discussions were engaged with by users 1773 times. A total of 86 Twitter profiles spanning 22 US states and 6 countries contributed to discussions among participants and the authors. Participants from all training levels were present, ranging from students to faculty. Interactions among participants and the case moderators were most commonly driven by clinical reasoning, including hypothesis-driven information gathering, discussing the differential diagnosis, and data interpretation or organization. Of 71 respondents to the local survey, 29 (41%) reported having a Twitter account. Of the 29 respondents with Twitter accounts, 17 (59%) reported participating in the case discussions. Respondents agreed that case participation increased both their clinical reasoning skills (15/17, 88%) and clinical knowledge (13/17, 76%).

**Conclusions:**

A social media–based serialized case discussion was a feasible asynchronous teaching method for engaging web-based learners of all levels in a clinical reasoning discussion. Further study should examine what factors drive trainee participation in web-based case discussions and under what circumstances asynchronous discussion might be preferred over in-person teaching activities.

## Introduction

Most training programs use a form of case-based discussion, termed “morning report,” to teach clinical reasoning to trainees [1,2]. The key feature of these conferences is interactivity [1,2]. Faculty facilitators lead case discussions that challenge trainees’ clinical reasoning while also teaching a framework for dissecting a clinical problem [2]. Increasing clinical demands and more recent needs for social distancing in the era of COVID-19 present barriers to attendance at long-form teaching sessions, suggesting that asynchronous teaching methods may be beneficial to trainee education [3]. Published interventions to present case-based teaching on the internet have used blogging platforms to disseminate clinical pearls [4,5]. However, because blogs generally serve as knowledge repositories or 1-way commentary on a specific topic, these interventions offer little opportunity for interaction among learners and teaching faculty [4].

Physicians and other health professionals increasingly use social media (SoMe) for medical education [6-11]. Twitter has emerged as a dominant SoMe medical education platform [6,7]. Professionals use Twitter to discuss research, network with colleagues, and disseminate educational material [6-8]. Dialogue among users is encouraged, and teaching can occur asynchronously. Students can access educational content at any time and place rather than synchronously through live content delivered in-person or by video broadcast [6].

Despite SoMe’s popularity among physicians for teaching and learning, few educational interventions have been published that use SoMe. Topf et al [10] published a Twitter-based adaptation of the nephrology journal club, and Lamb et al [11] designed a gamified surgical in-training exam study tool. A case-based teaching method on SoMe with sustained and tailored interactivity has not been previously described [6]. To fill this educational gap, we designed and implemented a web-based, asynchronous clinical case discussion series on the Twitter SoMe platform. The goals of this study were to assess the intervention’s global uptake on SoMe, examine the nature of interactions among web-based case discussion participants, and assess local attitudes regarding the intervention.

## Methods

### Setting and Participants

From February 2018 to August 2018, we developed a dedicated Twitter profile, @MedEdPGH, to host asynchronous case discussions on a biweekly basis. The project was advertised to internal medicine residents (n=182) and faculty (n=165) at the University of Pittsburgh Medical Center through email. Participation in the discussions was voluntary. In order to maximize web-based engagement, the SoMe account was made “public” so that any Twitter user, including those not associated with our institution, could view and participate in the content.

### Intervention Design

Case details were published on Twitter serially, with the history of present illness published first, followed by the examination, labs, and radiology findings. The first post in each case was introduced with a brief clinical vignette followed by the question, “What else do you want to know?” Case moderators replied to questions and provided subsequent aliquots of information at spaced intervals to encourage hypothesis-driven inquiry and discussion among participants. This format was chosen to mimic the incremental collection of information and cyclical clinical reasoning process clinicians use when seeing real patients [12,13]. When appropriate, the moderator encouraged participants to explain their reasoning or support their conclusions, as typically occurs in synchronous reasoning-centric case discussions [1,2]. Cases were concluded within 12-48 hours from the initial posting. This timing was flexible, depending on the moderator’s schedule.

Cases were prepared by the moderators (CNM and MGS). No real patient details were used to protect patient privacy. Clinical images (eg, radiology and rashes) were obtained from public sources with appropriate attribution. Each case discussion contained a series of partially scripted teaching points that were modified to highlight clinical reasoning pearls from the discussion. Several example case scripts are included in Multimedia Appendix 1.

### Assessment Process

A 6-month postintervention survey was sent to the trainees and faculty at our institution. Respondents were asked about their participation in the Twitter-based case discussions. A 5-point Likert scale was used to assess participant attitudes.

Web-based engagement was measured using Twitter analytics, which are freely available from Twitter. This approach has been used in previous educational Twitter interventions [10]. Impressions (number of times a post is viewed) and engagements (including number of clicks, replies, likes, or retweets) were recorded for the initial post in each case discussion 1 week after publication to help gauge web-based reach and participation. Descriptive statistics were computed using Microsoft Excel.

The locations and training levels of participating Twitter profiles were tabulated using each profile’s publicly available description. The authors also estimated the amount of time spent preparing for and moderating each Twitter Report case to measure the general impact on their daily schedules.

Two reviewers (CNM and MGS) examined participants’ posts and qualitatively analyzed themes of interaction among participants. Special attention was given to demonstrations of core clinical reasoning skills [12,13]. The categorization scheme was refined iteratively. Disagreements were fully adjudicated by the reviewers. Responses were monitored for unprofessional behavior, including cyberbullying, disclosure of non–Health Insurance Portability and Accountability Act–compliant information, and vulgarity.

### Ethics Approval

This was part of a larger study of SoMe use and underwent institutional review board approval (IRB #PRO17120325). Informed consent for the survey was obtained electronically. Survey data were collected anonymously, and all data from SoMe were deidentified before analysis. During the study period, the @MedEdPGH profile description contained a disclaimer indicating that it was being used for research purposes. All screenshots obtained from Twitter-based case discussions were taken with the permission of the participating accounts and are presented in a deidentified manner. No compensation was offered for participation in either the survey or the web-based case discussions.

## Results

During the study period from February 19, 2018, to August 19, 2018, a total of 11 web-based case discussions were hosted on the dedicated SoMe profile. The cases were viewed 21,845 times (impressions: average 1985/case) and engaged with 1773 times (engagements: average 161/case). Impressions and engagements peaked at 8426 and 634, respectively, for the penultimate case. The number of followers of the account increased throughout the study period, from 0 to 419. The largest increases in followers were seen on the days following each case discussion.

A total of 86 unique SoMe accounts spanning 22 US states, 2 Canadian provinces, and 6 countries (the United States, Canada, the United Kingdom, Australia, Malaysia, and El Salvador) contributed to case discussions during the study period. Of these, 8 were medical students, 21 residents, 29 attending physicians, 2 nurses, and 2 pharmacists. The remaining 24 profiles did not self-identify. Of these participants, 25 (29%) were from our organization. The average number of actively contributing profiles per case increased throughout the study period, from an average of 5 during the first 4 cases to an average of 18 during the last 4 cases.

**Figure 1:**
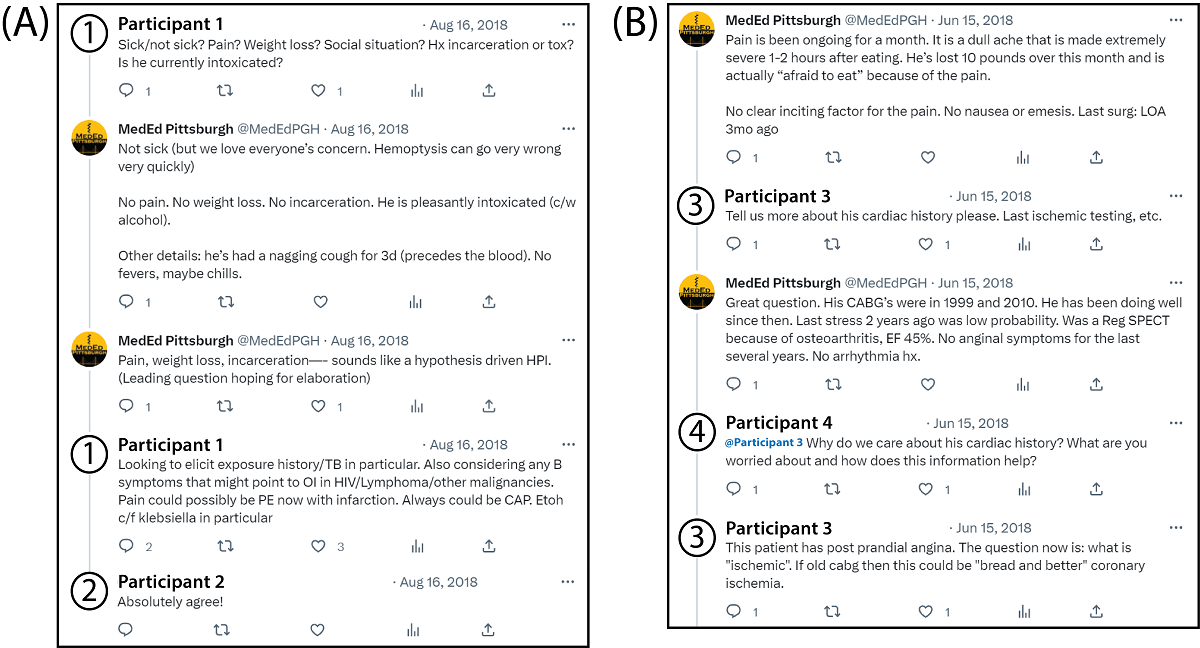
Sample Twitter case discussion interactions. All screenshots were taken with permission from the participating accounts. Their identities have been replaced with participant 1, participant 2, participant 3, and participant 4. (A) Participant 1 responds to our prompt, “What else do you want to know?” by conducting a hypothesis-driven inquiry about a case of hemoptysis. They elaborate on their differential diagnosis when pressed to explain their reasoning, and participant 2 seconds the differential. (B) Participant 3 asks for a more detailed past cardiac history of a patient with abdominal pain. When pressed by participant 4 for an explanation, participant 3 backs up their question with their differential diagnosis.

Participants generated a total of 242 posts throughout the study period. Posts in the discussion threads fell into several distinct categories. Clinical reasoning activities encompassed 7 categories: hypothesis-driven data collection (118/242, 49% of posts), elaboration of differential diagnoses (74/242, 31%), data interpretation and organization (31/242, 13%), sharing knowledge and schemas (29/242, 12%), discussing cognitive bias and metacognition (15/242, 6%), suggesting treatments or interventions (12/242, 5%), and problem representation (7/242, 3%). Other cataloged interactions included collegial banter (54/242, 21%), tagging other accounts (12/242, 5%), and sharing scholarly references (10/242, 4%). Participants interacted with the authors and also with each other to discuss cases. We witnessed no unprofessional behavior. Sample posts showing interactions among authors and participants are included in Figure 1.

Cases required an average of 30-60 minutes of preparation, depending on their complexity. Case moderation time also depended on overall case complexity and averaged 30-90 minutes of total screen time.

A total of 56 (31%) trainees and 15 (9%) faculty responded to the local postintervention survey. Of these, 29 (41%) respondents reported having a Twitter account, and 14 (48%) of these reported at least daily use of Twitter. Responses to the postintervention survey questions can be found in Table 1. Web-based, informal comments from participants about the quality and educational value of the exercise were universally positive.

**Table 1:** Postintervention survey results^a^.

	Responses (n=17), n (%)				
	Strongly agree	Somewhat agree	Neither agree or disagree	Somewhat disagree	Strongly disagree
Increased clinical reasoning skills	3 (18)	12 (71)	2 (12)	0 (0)	0 (0)
Thread well organized	6 (35)	4 (24)	4 (24)	3 (18)	0 (0)
Increased clinical knowledge	4 (24)	9 (53)	4 (24)	0 (0)	0 (0)

^a^Of the 29 survey respondents who reported having a Twitter account, 17 reported interacting with our Twitter case discussions. Their responses to the following 3 survey questions are presented: “Please rate how much you agree or disagree with the following statements regarding the case discussion posts on the @MedEdPGH Twitter account: (1) Case discussions helped to sharpen my clinical reasoning skills; (2) case discussions were well organized and easy to follow; and (3) by viewing Twitter case discussions, I was able to increase my clinical knowledge.”

## Discussion

We implemented a case-based, serialized, asynchronous method for teaching clinical reasoning using SoMe. Participants practiced core clinical reasoning skills like hypothesis-driven inquiry and differential diagnosis generation, similar to in-person case discussions, without any additional prompting from the authors. This intervention reached a global cohort of users across multiple disciplines despite local advertising only. Local reviews of our intervention were favorable, with survey respondents reporting positive effects on their clinical reasoning skills and clinical knowledge. The total time spent preparing and moderating each case discussion was equivalent to the time required to prepare and moderate a typical 60-minute morning report.

The asynchronous nature of SoMe presented several advantages over synchronous sessions. The timing of cases was adaptable to the moderators’ schedules, and cases were spaced throughout the day to avoid patient care–intensive periods. These results mirror the results of Jameyfield et al [14] who showed that emergency medicine residents preferred asynchronous teaching activities over synchronous didactics with respect to convenience and work-life balance. Trainees may also have benefited from the interleaving of skill practice throughout their day [15]. Web-based platforms additionally promote social distancing practices. The potential risks of using this strategy include distraction from clinical duties, increased SoMe use while at work, and the loss of socialization through in-person teaching. For example, a study by Primack et al [16] has shown that more frequent SoMe use correlates with higher rates of perceived isolation among young adults aged 19-32 years. Additional studies should investigate whether SoMe-based education affects students’ and trainees’ mental health and feelings of social isolation similar to recreational SoMe use.

While asynchronous participation is more flexible for learners, it could also result in them engaging less deeply with the material. We observed an array of participation patterns, ranging from a single post to in-depth engagement from beginning to end. Further qualitative work will be needed to understand what drives participants’ engagement in web-based case discussions.

Our intervention and assessment have limitations. We used survey methods and Twitter Analytics to judge attitudes and reach. While 2 validated scoring systems for educational blogs exist [17,18], no validated tools for assessing web-based, SoMe-based educational interventions are available. Last, our survey was distributed locally and not on Twitter to avoid responses from automated accounts (“bots”). This prevented results contamination but limited our ability to measure the intervention’s full web-based impact. It also limited our assessment to those local survey participants who had Twitter accounts, of whom only 17 reported participating in our intervention.

We show that SoMe can be used to engage multidisciplinary learners in a clinical reasoning discussion. While this intervention was conducted on Twitter, its format could easily be recreated on any SoMe platform. Further study is needed to elucidate differences in educational outcomes between synchronous didactics and asynchronous teaching using SoMe platforms.

## Appendix

Multimedia Appendix 1 Several example case scripts.

## Abbreviations

SoMe: social media
