# Supplementary material for: Developing a Web-Based Asynchronous Case Discussion Format on Social Media to Teach Clinical Reasoning: Mixed Methods Study
Source: JMIR Med Educ. 2023 Aug 9;9:e45277. doi: 10.2196/45277 (PMC10448285; doi:10.2196/45277)
Supplement: Multimedia Appendix 1 [file mededu_v9i1e45277_app1.docx]

**Supplemental Appendix 1 – Example Twitter Report Cases**

**Twitter Report Example 1**

**Alcoholic Rhabdomyolysis**

**1/**

Hey #MedTwitter friends! We’re back after a hiatus. It’s time for #TwitterReport

We give the case, you ask questions, everyone learns.

Case: A 60yoM w/ cirrhosis presents w/ low back pain and difficulty walking.

What else do you want to know?

**2/ #TwitterReport**

PMH: EtOH cirrhosis, CAD on ASA/Plavix (PCI 2 years ago)

SocHx: Former heavy alcohol use. Last use ?6 months ago? Smoke 1ppd.

Meds: Atorvastatin, ASA/Plavix, metop, lasix/spironolactone

Cirrhosis hx: No varices, +ascites on diuretics, no prior PSE, neg HCC screen

**3/ #TwitterReport**

HPI: 1 week of progressive pain in lower back+legs, progressive swelling in legs

2d of weakness of bilateral legs, can’t walk

- saddle anesthesia, loss of bowel/bladder control, numbness

+ dark-brown urine for 1 week

- abd pain, f/c/n/v

+ recently started drinking heavily again

**4/ #TwitterReport**

What will you be looking for on exam? In the labs (yes, we’ll give you LFTs)…

Here’s a poll to get the juices flowing:

You most want to examine for....

Ascites (POCUS!)

Lower ext strength exam

Lower ext sensory exam

Other (comment plz!)

**5/ #TwitterReport**

Exam: T 36*C, HR 78, BP 100/60, SpO2 95% RA

+Scleral icterus

RRR, nl S1/S2

CTAB, nl WOB

Abd soft, NT, +distention

POCUS: +ascites

LE’s swollen and tender, 4/5 strength limited by pain, 2+ reflex

LE sensation intact, normal saddle sensation, nl rectal tone

No asterixis

**6/ #TwitterReport**

Na 126, K 4.4, Cl 95, HCO3 20, BUN 50, Cr 5.9 (b/l 0.8)

WBC 8.1, Hb 13, Plt 82

TB 4.3, DB 2.7, AST 1102, ALT 472, ALP 211

UA -LE/Nit, ++blood, ++urobil, -protein, 0 WBC, 0 RBC

MRI: -cord badness

What are the top 3 things you are worried about for the LFTs? The AKI?

**7/ #TwitterReport**

For the LFTs: Some folks are thinking about Alcoholic Hepatitis... But!

>AST&ALT = too high. AlcHep usually = LFTs in low 100s

Pearl: When you have discordant data, stop. Ask if your 1* dx is right!

What else could be causing the elevated LFTs? Comment your thoughts!

**8/ #TwitterReport**

For the AKI, I know you want to spin the urine

>Urine microscopy = granular casts

But what about the urinalysis?

>UA with ++blood but 0-3 RBCs (that’s discordant!)

Now what do you think your AKI ddx should include?

**9/ #TwitterReport**

You guys are so smart! Here’s a recap:

>When I see discordant data, I SLOW DOWN. This stops my natural semmelweis reflex to reject the observation and prematurely close.

>Slow down by asking WHY! Think of the physiology and reason why the data doesn’t make sense.

**10/ #TwitterReport**

Case in points:

>When does AST>ALT? -- Cell death: liver ischemia, MI, muscle damage

>How can a UA show blood but no RBCs? -- “Blood” is heme. Heme without RBCs could be hemolysis, cell death (myoglobin from muscles), etc.

*******The picture begins to sharpen.

**11/ #TwitterReport**

One final trick when I’m stuck: Make a summary statement, even a formulaic one.

>Example: Leg weakness/pain + AKI + UA with heme without RBCs + AST>ALT

The patient has **Rhabdomyolysis**!

>> CPK = 20,000

So what do you think the Rhabdo is from?

**12/ #TwitterReport**

Rhabdomyolysis breaks down into

-Exertional

-Non-exertional, Traumatic

-Non-exertional, Non-traumatic >> Our patient is here.

NENT rhabdo is

-Infectious (virual, bacterial)

-Autoimmune

-Metabolic (genetic issues, HypoPO4, HypoK, etc.)

-Toxic

A common toxin: Alcohol!

**13/ #TwitterReport**

What about the Tbili and the ascites you say?

>>I agree x2. The patient also has experienced alcohol-induced decompensation of cirrhosis. Cirrhosis decompensation can’t explain the AST&ALT, but alcohol rhabdo can’t explain the Tbili.

**14/ #TwitterReport**

Case resolution:

-Patient is given albumin for possible HRS and also fluids are run for Rhabdo.

-The CPK downtrends, the Cr slowly returns to normal.

-The LFTs downtrend fast initially, but then more slowly

-Patient eventually discharged feeling better

**15/ #TwitterReport**

Final diagnosis: Alcohol-induced Rhabdomyolysis + Decompensated Cirrhosis

End.

Questions?

------------------------------------------------------------------------------------------------------------------------------------------

**Twitter Report Example 2**

**Panhypopituitarism**

**1/** Welcome back folks! #Twitter Report is back again on our biweekly schedule. We provide the case, you ask questions, everybody learns!

Case: An 80yoM with metastatic lung cancer p/w DOE and leg swelling. (PMH below in 2/ & 3/)

What else do you want to know? #MedTwitter #Tweetorial

**2/ #TwitterReport**

PMH: Extensive stage small cell lung ca, COPD, HTN, HL

PSH: right hip ORIF after traumatic fx, 1y ago

FH: healthy(!)

Soc: quit smoking 20y ago, 50ppy; no EtOH

Medications: Albuterol, Amlodipine, Atorvastatin, LAMA/LABA, Metoprolol, Hydrocort cream

**3/ #TwitterReport**

Great questions! Let me start with a teaching point. When you have a person with a complex, chronic illness history >> know that history. Why?

1) It’s a part of their personal story.

2) Whether it’s the Dx, it shapes how they experience their present issue.

**4/ #TwitterReport**

Lung SCLC Hx:

-Dx with SCC 9mo ago after nodule found on screening CT

-Chemo: Etoposide/cisplatin + atezolizumab >> atezo maintenance for last 3 months

>>Only side-effects from atezo were low-grade dermatitis (on steroid cream) and subclinical hyperthyroid

**5/ #TwitterReport**

More teaching: Immune-related adverse effects (irAEs) are not uncommon with PD1/PDL1 inhibitors.

>Dermatitis in 10-30%. Rx topical steroid.

>Thyroiditis in 10-20%. Usually sublinical. If so, get regular TSHs until they --> hypothyroid. Then give synthroid.

**6/ #TwitterReport**

HPI: (you mean you don’t have the dx yet? 😊)

-Patient at baseline – ambulating well with good energy – until 2wk ago when he noted slowly worsening DOE.

-Now, can only ambulate across room before he gets SOB.

>Denies f/c/n/v/d, cough, sick contacts, chest pain.

**7/ #TwitterReport**

HPI cont:

-Also reports overwhelming fatigue. Sleeping 15h/d.

-New L leg swelling for last 3d.

Okay! Give me your problem list and 3 Ddx concerns. Is this all a single etiology or are there multiple issues here?

Single issue: Occam’z razor

Multi-issues: Hickam’s dictam

**8/ #TwitterReport**

Let’s see how those Ddx change w/ Exam + Labs:

VS: T96.6, BP 102/63, HR 103, SpO2 96%

GEN: NAD, appears tired

HEENT: MMM, no LAD

CV: tachy, regular, nl S1/S2, 2+ LLE edema to mid-shin

Pulm: CTAB, nl WOB

GI: Soft, NTND

Derm: no rashes

Neuro: 5/5 strength, AaOx3

**9/ #TwitterReport**

WBC 7.4 (PMN 59%, Eos 5%), Hb 12, Plt 215

Na 141, K 3.7, Cl 104, HCO3 23, BUN 18, Cr 1.4 (b/l 1.1), Glu 97, Ca 9.4, Alb 3.8

LFTs normal

BNP 212

Tn normal

From 20d ago: TSH 0.05, Free T4 1.2

EKG: sinus tach, rare PVCs

CXR: clear

Has your ddx changed? What do you want now?

**10/ #TwitterReport**

Interesting thoughts, everyone!

A venous duplex is obtained: Acute DVT of left leg from calf to iliac vessels --> start heparin gtt.

His Cr on day 2 is 1.1. Could a PE explain his symptoms? Would you get a CTA? Poll below, but explain your reasoning please!

Explains sx, Yes CTA

Doesn’t explain, Yes CTA

Explains sx, No CTA

Doesn’t explain, No CTA

**11/ #TwitterReport**

At the time, a PE was thought to explain his DOE, but not the fatigue, per se, which could have been deconditioning or his PD1 inhibitor. A CTA was obtained...

CTA: Cancer is unchanged. No PE but a subsegmental one could have been missed.

Where to now?

Echocardiogram

VQ Scan

Infection w/u

Other (explain!)

**12/ #TwitterReport**

Advancing the case:

Hosp Day #3: BP 90/60, HR 62

Somnolent but arousable, LLE edema improved

Heart, Lungs, Abd unchanged and normal

CBC unchanged. BMP shows Na = 128 now. LFTs still normal.

>BCx are sent; CXR still clear

>repeat TSH 0.001, Free T4 0.8

**13/ #TwitterReport**

Patient is borderline hypotensive, somnolent. He’s hypoNa now. TSH still low, Free T4 still normal

>>What do you think is going on here? What are your ideas?

>Poll: Are these new problems, or a new view of the original issue? Explain your reasoning!!

New problems!

Old problems, new view!

**14/ #TwitterReport**

When I’m stuck, I summarize a case --> 80yoM with Lung SCC on PD1 inh with presumed ICI-induced thyroiditis p/w fatigue, DOE and develops hypotension, hyponatremia, and decreasing TSH & FT4.

Question is, does this fit anyone’s illness scripts? Or ring any bells?

**15/ #TwitterReport**

Given the new hyponatremia, decreasing thyroid studies, and hypotension, there was concern for hypopituitarism.

8AM cortisol = 1, ACTH = low, stim was borderline normal --> central adrenal insufficiency

>He is started on steroid replacement.

**16/ #TwitterReport** #TipsForNewDocs

***If you are worried about adrenal crisis, snag a cortisol and an ACTH, then give dexamethasone.

> Dex doesn’t interfere with the cortisol assay. Can still do a stim test

> Severe hypotension is often a good enough “stim test” of its own.

**17/ #TwitterReport**

His other pituitary labs are off as well:

- LH, FSH low

- Insulin-like growth factor, low

- Prolactin borderline low

MRI brain: not obtained b/c it wouldn’t change management

Final Dx: Immune Checkpoint inhibitor hypophysitis --> panhypopituitarism

**18/ #TwitterReport**

Great work on a difficult case, everyone! I’ve sprinkled the teaching throughout the case, so I’ll skip any teaching here at the end. Hope you learned something new!

See you in 2 weeks for the next report!

------------------------------------------------------------------------------------------------------------------------------------------

**Twitter Report Example 3**

**Marantic Endocarditis**

**1/** Welcome back to #TwitterReport. We give you a case. You ask questions. Everybody learns!

Something different today: I’m giving you the whole ED w/u (they did a good job). You pick up where Medicine picks up.

Case: Admission for stroke w/u.

***Questions start on page 4!

#MedTwitter

**2/ #TwitterReport**

Story: 71yoM with mets lung adeno ca. (liver, spine mets) p/w

-2wk eye blurriness w/ subtle R field cut on exam

-2d confusion, lethargy, apraxia

MRI: multiple, small acute-subacute infarcts in R>L cerebral lobes

CTA H&N: patent large vessels

EKG: normal sinus rhythm

**3/ #TwitterReport**

PMH: CAD, BPH, HTN, HL, OSA

-New lung adeno ca diagnosis 3mo ago, extensive mets

Meds: ASA, amlodipine, atorvastatin, carvedilol, lisinopril, tamsulosin

FH: Father with strokes, mother with lung ca

**4/ #TwitterReport**

Basic Exam: T37.1, BP 140/81, HR 67, SpO2 95% RA

>Heart, Lungs, Abdomen clear/normal

Neuro: Apraxia and R field cut as above

Basic Labs:

WBC 13, Hb 13, Plt 130

BMP nl (Cr 1.1 is b/l), LFT nl

>What else do you want to know?

>Where do you think the strokes came from?

Lacunar

Atherothrombotic

Cardioembolic

Other (explain)

**5/ #TwitterReport**

***The history helps you get to 80% of diagnoses. Let’s go thru each choice...

>Lacunar: the radiologist will usually call this on the MRI read. Multiple bilateral lacunae is also odd

>Atherothrombotic: Would typically be one territory. CTA may show the thrombosis.

**6/ #TwitterReport**

>Embolic: sudden onset, hemorrhage common. Can be 1 or multiple territories

>>Bigger territories are bigger --> higher chance of receiving emboli

---Thus, MCA territory = most common; but any is possible

So: Embolism = most likely but we must find a source!

**7/ #TwitterReport**

In the search for a cardiac source, would you get a TTE or go straight to a TEE? A poll is below. As always, explain your reasoning in the comments!

TTE

TEE

**8/ #TwitterReport**

TEE is more sensitive for detecting cardioembolic sources: <https://pubmed.ncbi.nlm.nih.gov/1987242/>

The decision here is instead about *Pretest Probability*

>Our pretest probability is HIGH --> A negative TTE = suspected false negative

So: TEE 1^st^ if you can

**9/ #TwitterReport**

TEE is obtained: No LV or LAA thrombus, LVEF 55%

>> two 20mm vegetations on the mitral valve

>> Mild MR

Here is our source of embolism. BCx are obtained.

Fast-forwarding: now after 3 days, BCx remain negative. No fevers. WBC still ~12-14.

What are the veg’s from?

**10/ #TwitterReport**

1^st^: Culture negative endocarditis = 7d of negative cultures, at least 3 separate blood samples.

What are the most common causes of culture negative endocarditis?

HACEK organisms

Fastidious organisms

Cancer

Antibiotics

**11/ #TwitterReport**

The answer is antibiotics! Usually, Strep species killed by prior Abx.

What about Haemophilus, Actinobacillus, Cardiobacterium, Eikenella, Kingella (HACEK) from Step 1?

>>These usually grow <5d w/ modern culture technique! <https://pubmed.ncbi.nlm.nih.gov/16267743/>

**12/ #TwitterReport**

When should I think about fastidious organisms? --> Go back to the history!

Farms w/ animals: Coxiella, Brucella, T. whipplei

Lice, homeless: Bartonella quintana

Cats (Kittens): Bartonella henselae

Immunesuppression/Lines: Fungus

**13/ #TwitterReport**

Our patient has a completely negative exposure history and no B-symptoms, joint sx, GI sx, respiratory sx

--Further testing is deemed low yield.

With completely negative cx, he is finally dx with marantic endocarditis (nonbacterial thrombotic endocarditis).

**14/ #TwitterReport**

NBTE presents a very high embolic risk. Rx = treat cancer + anticoagulate, whether there is e/o stroke or not, as long as there is no contraindication.

Our patient does well on anticoagulation and is eventually discharged with a plan for chemo.

**15/ #TwitterReport**

Final Dx: Embolic strokes from marantic endocarditis a/w metastatic lung adenocarcinoma.

We hope you learned something today and yesterday! Give us your feedback in the comments below!

------------------------------------------------------------------------------------------------------------------------------------------

**Twitter Report Example 4**

**Euglycemic Diabetic Ketoacidosis**

**1/** Welcome back to #TwitterReport. This week we have a short case for you. Buckle up: it is jam-packed with learning!

Case: A 40yoF p/w diffuse abd pain, severe SOB, and worsening lethargy. Labs shown. HR 120, BP 140/90, RR 33, SpO2 98%.

What else do you want to know?

TR 081920 (Picture 1).jpg

**2/ #TwitterReport**

You guys are asking all the right questions. Before I get into the history, PMH, and meds, let’s go over the labs themselves.

***Step 1: Determine what the –emia and –osis are.

-pH is low = acidemia

-Bicarb is low w/ acidemia = metabolic acidosis

**3/ #TwitterReport**

***Step 2: Determine the anion gap.

Na – Cl – HCO3 = AG

150-111-3 = 38(!)

Generally: any gap above 20 should make you nervous.

>>A pH<7.2 should also make you nervous, so you were likely already in high gear.

**4/ #TwitterReport**

***Step 3: Assess delta-delta (no division necessary!)

>How much is HCO3 decreased from normal: 24-3 = 21

>How much is anion gap increased from normal: 38-13 = 24

--> 21 and 24 are within 6 mmol/L of each other, so I am less concerned for a second metabolic process

**5/ #TwitterReport**

>>The 6 mmol/L fudge factor comes from buffering of acids (the AG) by the bone and not just serum HCO3.

*If the delta-AG had been << the delta-HCO3, I’d have been worried about non-gap acidosis (I.e. the HCO3 is decreased more than the AG can account for).

**6/ #TwitterReport**

Step 4: Assess compensation (generally respiratory):

>Winter’s formula: expected pCO2 = 1.5*HCO3+8 (+/-2)

> 1.5*4+8=14 --> She has appropriate respiratory compensation

So we have a whopping, pure anion gap metabolic acidosis w/ respiratory compensation!

How come?

**7/ #TwitterReport**

This is the only mnemonic I remember from all med school: GOLDMARK

Gap Acidosis Ddx--

G: glycols

O: oxoproline (a Tylenol metabolite)

L: L-lactate

D: D-lactate (gastric bypass patients)

M: methanol

A: Aspirin

R: Renal failure

K: Ketoacidosis (diabetic, starvation, alcohol)

<https://www.thelancet.com/pdfs/journals/lancet/PIIS0140-6736(08)61398-7.pdf>

**8/ #TwitterReport**

To suss out a cause, we need a history (from family):

-Feeling well until 6d ago when she developed diffuse abd pain, minimal N/V.

-2wk prior, she started a new medication which “made her pee a lot more”

PMH: Diabetes on insulin and pills, HTN

**9/ #TwitterReport**

Family says she didn’t take insulin last 3d because sugars were only ~200 and she wasn’t eating.

>They don’t have a medication list or know what the new medication was...

>No history of alcohol or drug use. A drug screen is negative

Any ideas yet?

**10/ #TwitterReport**

One more aliquot will do it, I think. A search for infection is started and a UA is obtained:

SpGr 1.024

Blood 3+

Ketones 3+

LcE 2+

Nitrite 1+

Protein 100

Glucose >1000

Squams lots and lots

WBC 26

Bacteria none

Also, serum lactate = 2.2

What’s the diagnosis?

**11/ #TwitterReport**

Always look for the discordant data! Our patient had a serum glucose of 208 and a UA shows +++ glucose.

The usual renal resorption threshold for serum glucose is ~180. That’s way too much urine glucose for such a weakly high serum glucose!

**12/ #TwitterReport**

Pharmacy helps to confirm: New Med = an SGLT2 inhibitor!

>Therefore, a summary shows us: 40yoF with DM recently started on SGLT2i who develops

Final diagnosis = euglycemic DKA from SGLT2i use

**13/ #TwitterReport**

Euglycemic DKA occurs with serum glucose <300. It is fairly rare: ~0.1% w/ SGLT2i use.

SGLT2i’s predispose to EuDKA for two reasons:

-Increased glucosuria reduces serum glucose levels

-SGLT2i’s enhance ketogenesis

<https://care.diabetesjournals.org/content/38/9/1638>

**14/ #TwitterReport**

Thanks for following along! We hope you learned something. Come back in another two weeks for another rousing round of #TwitterReport!

------------------------------------------------------------------------------------------------------------------------------------------

**Twitter Report Example 5**

**Pulmonary Alveolar Proteinosis**

**1/** Welcome to #TwitterReport! We give you a case, you ask questions, everybody learns! Today’s patient presents to clinic, and he’s short of breath

Case: 30yoM w/ asthma p/w 3mo of worsening cough, SOB despite abx+steroids x2 via urgent care.

What else do you want to know?

**2/ #TwitterReport**

PMH: asthma (never hospitalized)

FH: no lung disease, lupus in mother, hypothyroid in sister

Soc: daily tobacco smoking, occasional marijuana smoking, works a desk job

Meds: Albuterol inh (uses rarely)

Any clarifying questions about past histories?

**3/ #TwitterReport**

HPI: Began 3mo ago with a nonproductive cough --> very mild DOE --> DOE + SOB at rest.

>2mo ago, got abx+steroids for “PNA” (CXR normal). Felt a little better, never all better

>2 weeks ago, got much worse: abx+steroids helped a little.

>Worse again, so --> clinic

**4/ #TwitterReport**

ROS: Denies fevers, chills, night sweats, changes in weight, orthopnea, PND, sick contacts

>Does admit to vaping occasionally. Uses commercial cartridges, nothing bought on the internet and nothing with CBD

**Exam and a CXR next. Let me know what else you want!

**5/ #TwitterReport**

T98.6*F, HR 90, BP 110/70, SpO2 86% RA, RR 18

HEENT: conjunctiva pink, OP clear

Cards: RRR, no mrg, no edema, JVP nl

Lungs: fine rales at bases, nl WOB

Abd: soft, NTND

Neuro: AaOx3, 5/5 strength

Chest xray: Tell me your read! What do we do now (he’s in clinic!)?

[TR 090220 CXR1.jpg]

**6/ #TwitterReport**

1) He’ll go to the ER on O2

2) This chest x-ray shows a diffuse, basilar > apical interstitial pattern. In someone with chronic, progressive, pulmonary complaints, we should worry about Interstitial Lung Disease.

>A CT scan is warranted

How will you order the CT?

With contrast

Without contrast

High resolution

**7/ #TwitterReport**

Of course, if you want to see the interstitium, you need high res. How is a HR CT different from a usual CT?

>It takes 1-2mm cuts spaced 10-40mm apart

***HR CTs don’t image the entire lungs, so you can miss small nodules, cancers, etc.

>>Choose imaging wisely!

**8/ #TwitterReport**

Here are the images, courtesy of Radiopaedia, to protect patient confidentiality.

So what do you see? Are we satisfied with calling this ILD? Is that a leading question? What is your ddx?

[TR 090220 CT1.jpg]

[TR 090220 CT2.jpg]

[TR 090220 CT3.jpg]

[TR 090220 CT4.jpg]

**9/ #TwitterReport**

What you see on these CT images is called “crazy paving,” which is a combination of interlobular septal thickening and ground glass.

Anyone have a differential diagnosis for Crazy Paving? (before I show one)

**10/ #TwitterReport**

Crazy Paving is nonspecific, but a few things classically display it:

-ARDS, including acute interstitial pneumonia (idiopathic ARDS)

-Pulmonary alveolar proteinosis (very rare)

Many other things “can” show crazy paving: aspiration, TB, PJP, drug pneumonitis, DAH

**11/ #TwitterReport**

Radiopaedia has a good list for you guys to peruse: <https://radiopaedia.org/articles/crazy-paving?lang=us>

Basic labs are normal.

So what should we do next for our gentleman, now stable on 4L of O2, comfortable in bed? Antibiotics

Bronch

Steroids

Autoimmune Labs

**12/ #TwitterReport**

Taking the answers in turn:

>Abx: He’s stable and we don’t know what we’re treating. Let’s hold off.

>Steroids: same as for Abx.

>Autoimmune labs: why not

***Bronch: this is where the $$$ is. It will give us cells, cultures, and histology if they take bx.

**13/ #TwitterReport**

Other Labs: ANCA, ANA, RF, anti-GBM negative. Myositis panel pending

Bronch results: lavage fluid is milky/opaque. Microscope: lots of PAS positive, granular proteinaceous material concerning for PAP

Serum antibodies for GM-CSF are present.

**14/ #TwitterReport**

Final Diagnosis: Pulmonary Alveolar Proteinosis

Summary points:

-Ask about vaping! What they vape and where they purchased (internet?)

-HRCT is good for ILD but not great for looking at entire lungs!

-Crazy paving is GGOs + septal thickening. Has its own DDx

------------------------------------------------------------------------------------------------------------------------------------------

**Twitter Report Example 6**

**Metastatic Testicular Cancer – Cannonball Mets**

**1/** It’s time for #TwitterReport and we’ve got a case with your name on it! We provide the case, you ask questions, everyone learns.

Case: Healthy 24yoM presents with SOB worsening for the last month.

Similar to the last report, let’s see how far we get just on the ED signout...

**2/ #TwitterReport**

Here’s your signout:

24yoM, no PM Hp/w worsening SOB for a month

>Fevers on an off. Presently febrile to T100.5*F

>Also hypoxic to 88% on RA, up to 94% on 2L, not using accessory muscles

>CXR looks nasty (credit Radiopaedia)

>Got BCx and gave CTX+Azithro for PNA

[TR 092320 CXR1.jpg; TR092320 CXR2.jpg]

**3/ #TwitterReport**

He’s coming to your service. What else do you want to know? What is on your ddx so far?

**4/ #TwitterReport**

Great questions. Some of you have the answer already!

HPI: as above, but also...

>15# unintentional weight loss over 2 mo

Has night sweats, nausea, nonproductive cough, no LE edema

>Not sexually active, never checked for HIV

Ddx thoughts? Where to focus your exam?

**5/ #TwitterReport**

Great thoughts again. The exam, abridged:

T98*F, HR 85, BP 120/80, RR 18, SpO2 96% 2L

Lungs: rhonchi throughout

CV: RRR, no mrg, no edema, nl JVP

Abd: soft nt/nd, no hsm

GU: large right testicular mass(!)

Know the dx yet???

**6/ #TwitterReport**

Before we reveal the final dx, let’s talk about that CXR. I’ve included it again for reference. Credit goes to Radiopaedia. And now, a poll!

What pattern would you describe this CXR with? If the answers don’t work for you, comment your own description:

Alveolar pattern

Interstitial patern

Lobar pattern

Nodular pattern

**7/ #TwitterReport**

Let’s take these answers in turn:

>Alveolar pattern: from fluid filling up alveoli

--appears fluffy and has indistinct edges (poorly marginated)

--if related to pulmonary venous congestion, may be centered around the hila

[TR 092320 CXR3.jpg]

**8/ #TwitterReport**

>Interstitial pattern: from fluid in the interstitium

--can appear as lines (linear), as a mesh/net/lace (reticular), as small nodules (nodular), or a combo (reticulonodular)

A great resource on linear vs. Alveolar patterns: <http://www.wikiradiography.net/page/interstitial+vs+alveolar+lung+patterns>

[TR 092320 CXR4.jpg]

**9/ #TwitterReport**

>Lobar pattern: a consolidation in a lobe of the lung

--homogeneous opacification of an entire lobe or a segment

>>may have sharp lines delineated by a fissure

--bronchi still has air --> air bronchograms

--unlike atelectasis, there is no associated volume loss

[TR 092320 CXR5.jpg]

**10/ #TwitterReport**

>Nodular pattern – our patient has multiple, large, well-circumscribed nodules (ie, masses)

For subacute worsening SOB with B-symptoms and large pulmonary nodules, a Ddx could be:

-metastatic cancer

-Indolent infection (Histo/Blasto)

-?Autoimmune?

**11/ #TwitterReport**

With a testicular mass... These well-circumscribed masses have a special name: Cannonball Metastasis

Testicular cancer and Renal Cell Carcinoma are classic causes.

Check out this article from Radiopaedia: <https://radiopaedia.org/articles/cannonball-metastases-lungs?lang=us>

**12/ #TwitterReport**

Here’s how the case plays out:

>CT Chest/Abd/Pelvis w/ contrast: Lots of nodules, lots of lymphadenopathy, Liver metastasis present

>Brain MRI w/ contrast: No metastasis

LDH: 1135

AFP: 85 (nl)

B-HCG: 60,000

Based on these labs, which type of testicular ca is most likely:

Embryonal

Lymphoma

Seminoma

Yolk Sac

**13/ #TwitterReport**

Tumor markers are not specific; you need histology, but...

>B-HCG or AFP is only >10,000 in germ cell tumors

>LDH is nonspecific

>AFP is not elevated in Seminoma

>AFP is elevated in Yolk Sac tumors

So our $ is on Embryonal (or choreocarcinoma)

**14/ #TwitterReport**

Back to the case:

>Undergoes orchiectomy (never biopsy! Can generate metastasis!)

--Path = Mixed Germ Cell Tumor: 70 embryonal, 20% choreo, 5% seminoma, 5% yolk sac

>Gets VIP (etoposide, ifosfamide, cisplatin)

>Frequent tumor lysis labs b/c of extent of disease

**15/ #TwitterReport**

He tolerates chemo well (needs some GM-CSF for neutropenia) but completes treatment and is disease free 1 year out! (Of course, all details are changed to protect our patient, but they are doing well).

Thanks for following along! See you next time!

------------------------------------------------------------------------------------------------------------------------------------------

**Twitter Report Example 7**

**Coxiella Septic Arthritis**

**1/** We’ve got you back in clinic today for an exciting #TwitterReport. We give you a case, you ask questions, everybody learns!

CC: Look at my wrist!

Case: 70yoM p/w 3d of fevers and left wrist pain/swelling

What else do you want to know? (Histories below; image via Medscape)

**2/ #TwitterReport**

PMH: HTN, HL, ankle OA

PSH: Bilateral prosthetic shoulders and knees

>Got steroids injected to ankles 2 weeks ago

Soc: Cattle farmer, went to cattle auction 2 weeks ago, drinks his cows’ milk

>Denies smoking, alcohol

>Sexually active w/ wife only

Meds: atorvastatin

**3/ #TwitterReport**

Good questions. I’ll move to the HPI and exam together

HPI: Issues with B ankle pain for years. Got steroid inj for 1^st^ time 2 weeks ago w/ pain relief

-Well until 3d ago: had night sweats + daytime fevers --> left wrist b/c swollen, painful. Can’t bend wrist

**4/ #TwitterReport**

HPI cont:

-no h/o trauma, cuts

-7d ago, right elbow hurt, but that’s better now

ROS: denies other joint pain/swelling, dysuria, penile d/c, rash, eye pain, vision changes, back pain

**5/ #TwitterReport**

VS: T37, BP 153/89, HR 79, SpO2 98%

HEENT: OP clear, no icterus

CV: RRR, no mrg, no LE edema

Lungs/Abd: nl

MSK: left wrist exquisitely TTP, red, swollen w/ effusion, no other joints affected

GU: No penile d/c

Labs:

-WBC 17k, PMN 80%, Hb 13, Plt 350

-Cr 1.2 (at baseline)

**6/ #TwitterReport**

Wrist X-ray: degenerative arthritis

BCx pending.

Summary: 70yoM farmer with recent ankle steroid inj p/w acute fevers, left wrist arthritis w/ effusion and leukocytosis.

What is on your Ddx?

What would you do next?

**7/ #TwitterReport**

Everyone here is c/f septic arthritis --> arthrocentesis. Pt sent to the ER for tap + abx. Results:

Fluid: pus

WBC 151,000, 95% PMNs

Gram stain: no organisms

No crystals

>Pt put on Vanc+CTX. To OR for I&D given purulence. In OR: more pus. OR Gram stain negative.

**8/ #TwitterReport**

Fast forward to day 3:

-wrist better s/p I&D

-new right elbow pain + swelling

-left ankle pain+swelling

-Cultures negative 48h

Elbow/ankle aspiration: pus, WBC ~100k, gram stain negative, no crystals

>New joints are involved! Thoughts?

Poll: I think the arthritis is...

Infectious

Autoimmune

Crystals

Put ddx in comments!

**9/ #TwitterReport**

So this is puzzling. A trick I use when I’m stuck is to rename my problems as much as possible. So, his “arthritis” becomes

>>Culture negative polyarthritis w/ WBC >100k

**There are many infectious and inflammatory/rheumatic causes of Cx neg polyarthritis! The WBC>100k pushes us towards infections. (Crystals also do this, but there have been none on the taps)

**10/ #TwitterReport**

So what infxns might a stain/culture miss from a joint?

-Viruses: CMV, EBV, Coxsackie, Parvo, HepB/C

-Fungus: Blasto (not immune compromised)

-Bacteria: Gonococcus, Bartonella, Brucella, Coxiella

T. whipplei, Lyme

>Risk factors: farmer, cattle, unpasteurized milk

**11/ #TwitterReport**

It makes sense to test for all of these, but which ones fit his risk factor profile best? Write down your list and check your answers on the next post!

(It may seem annoying to write stuff down, but this is the best learning tactic! Make it stick!)

**12/ #TwitterReport**

Answers:

-Outdoor exposure: Lyme

-Cattle/milk: Brucella, Coxiella, T. whipplei

-HepB/C, HIV, and GC worth considering on anyone (you never know)

>>Could also consider deeper infx of common organisms (IE, back abscess) but would expect BCx to be positive!

**13/ #TwitterReport**

Here’s how the case plays out:

>Abx narrowed to CTX/doxy, given above concerns

>BCx remain negative; AFB staining was negative also

>TTE and TEE show no vegetations

>Hep A-C negative, HIV neg

>Urine GC negative

>Lyme: only 2 bands (negative)

**14/ #TwitterReport**

More results:

>EBV, CMV, Pavro, Coxsackie titers negative

>Blasto Urine Ag negative

>Brucella, Bartonella titers neg

>Coxiella: Phase I IgM-, IgG+, Phase II IgM+, IgG+

--> This is consistent with an acute infection: Q fever!

The patient is switched to doxy only

**15/ #TwitterReport**

His symptoms of f/c/ns, arthritis disappeared (minus his chronic ankle issues, which were from OA).

>He took doxy for 6mo total.

>Convalescent serum showed *higher* IgG titers and disappearance of IgM --> yeah, this was Q fever

*He continues to do well w/o any symptoms.

**16/ #TwitterReport**

Final Diagnosis: Q fever with polyarthritis

Thanks for playing, everyone! We hope you learned something. See you in two weeks for another case!

------------------------------------------------------------------------------------------------------------------------------------------

**Twitter Report Example 8**

**Scurvy with Hemoptysis**

**1/ #TwitterReport**

We give you a case. You ask us questions. Everyone learns. (Big updates in this thread)

55yoM with prior and current heavy alcohol use presents with new onset hemoptysis for 2 days.

What else do you want to know?

**2/ #TwitterReport**

So our guy is rock solid stable. HR 90, BP 140/75, 99% on room air.

ABC’s are solid

He’s had 3d of coughing fits. No fevers, maybe chills.

Scant hemoptysis, no clots, No mouth or nose bleeding.

HTN in the past. No meds.

No chronic B-symptoms overall. No incarceration.

**3/** **#TwitterReport**

No bleeding history before. No smoking history. No cocaine or heroin.

Drinks 2-3 gal of alcohol per week. Says he doesn’t eat very well but gets enough calories from alcohol.

Looks kind of disheveled overall.

**3/ #TwitterReport**

The differential so far from the group:

TB, CAP, beriberi/alcohol CHF, cancer, opportunistic infection.

The case has also revealed underlying MDD and some suggestion of poor nutrition.

LFTs mildly elevated. Coags nl

Who wants labs and imaging? What kinds?

**4/ #TwitterReport**

CBC 8>14.4<200

BMP 140/3.4 105/24 10/0.8<90

Ca 9.0, Alb 3.9, Mg 1.7, PO4 nl

Lactate and LDH – please teach us why y’all wanted this and I promise I will tell you.

VitC and thiamine -pending…

CXR is completely clear

**5/ #TwitterReport**

CTA with contrast: No PE or infarct. No bronchial varices. Very small area of RLL ground glass that could be PNA (clinically correlate)

I’ll emphasize. PNA = very small. We didn’t think it large enough to cause the hemoptysis.

What says the group?

**6/ #TwitterReport**

Almost forgot, liver morphology is normal on that CT.

We love everyone’s thoughts. There’s diagnostic uncertainty here, is the issue. The dots don’t connect.

We think one participant summarized our thoughts on the current situation: nl labs and a fairly benign CT make “bad” stuff less likely in the s/o a well-appearing patient

**8/** **#TwitterReport**

Bronchitis and PNA are most common in this situation (we have e/o both in the HPI), but the lesion seems disproportionately small. Can the smallest of PNA’s cause hemoptysis?

If it can: Case closed.

If not, you’re left asking “why is his mucosa so friable?”

**9/** **#TwitterReport**

Case continued: Lactate nl. Patient feeling better on abx

Thiamine low normal. Vitamin C undetectable.

The question becomes: Hickum’s dictum or Occam’s razor

Aspiration(?) + coughing alone = hemoptysis?

Mild aspiration + mucosal friability = hemoptysis?

**10/ #TwitterReport**

Ultimately, this is probably unknowable.

But the diagnosis of scurvy was made because of the odd looking hairs (swan necks and corkscrews, probably), darkened gums, ?out of place hemoptysis, and hx of poor nutrition.

**11/** **#TwitterReport**

Either way, we remind ourselves to check our theories against the pathophysiology we see.

When the dots don’t connect, we ask “what could be missing?”

Final Diagnosis: aspiration +/- PNA with hemoptysis as a result of VitC deficiency

Thoughts?
